# Seed production system and adaptability of okra (*Abelmoschus esculentus* L.) cultivars in Buea, Cameroon

**DOI:** 10.1371/journal.pone.0278771

**Published:** 2022-12-14

**Authors:** Raymond Ndip Nkongho, Jill Tracy Efouba-Mbong, Lawrence Monah Ndam, Gwendoline Ashie Ketchem, Ebob Glacilia Etchu-Takang, David Tavi Agbor

**Affiliations:** 1 Department of Agronomic and Applied Molecular Sciences, Faculty of Agriculture and Veterinary Medicine, University of Buea, Buea, Cameroon; 2 Department of Food Science and Technology, Faculty of Agriculture and Veterinary Medicine, University of Buea, Buea, Cameroon; 3 Department of Agrochemistry and Soil Science, Faculty of Agronomy, Agricultural University Plovdiv, Plovdiv, Bulgaria; Gomal University, PAKISTAN

## Abstract

Okra is grown globally for its nutritional and economic benefits. Okra seeds ensure continuous production of the crop but challenges of poor production, adaptability and management may not allow the seeds to express their full potential. There are two seed production systems in Cameroon; the informal and formal. In Buea, the informal seed system is used by most farmers for seed production/utilization and farmers are reluctant to use hybrid seeds. This study aimed to assess the informal seed system of okra and evaluate the adaptability of seed produce from informal and formal systems in Buea. A survey and a field experiment were carried out. The designs for the survey and field experiment were stratified random sampling and randomized complete block design respectively. Data collection for the survey was done using questionnaires and other data collection instruments, while for the field experiment, data was collected on germination, vegetative growth parameters, incidence and severity of pests / diseases and yield. Data analysis for the survey was done using descriptive statistics, while data from the field experiment was done using a two-way ANOVA test and treatment means compared using the Tukey test at 5% probability. Results from the survey showed that women (60%) were mostly involved in seed production by mass selection from two landraces identified. Preservation of seeds was mostly done with the use of wood ash (58%) and insects were the major postharvest pest (76%). For the field experiment, at 66 DAP, Yellen recorded the highest significant number of leaves (13.417), leaf area (771.4 cm^2^) and the number of branches (5.64). Clemson spineless recorded the highest significant incidence (89.9%) and severity for pests / diseases while Kirikou and Landrace recorded the least incidence (0.0%) and severity. Kirikou recorded the highest significant yield (6.0 tons/ha), followed by Landrace (5.3 tons/ha). These findings reveal the performance of the landrace and provide reasons why farmers in Buea are reluctant to use hybrid okra seeds. The Landrace should certainly have adaptable genes, coupled with the autogamous nature of okra which encourages inbreeding for homozygous traits, which are dominant in expression compared to heterozygous traits.

## Introduction

Okra (*Abelmoschus esculentus* (L.) Moench), is an economically important annual vegetable grown from seed in tropical, subtropical and warm temperate regions [1, 2]. Vavilov [3], Murdock [4], Dantas, *et al*. [2], proposed the origin of okra in the Abyssinian centre. Okra later spread to the Caribbean and the U.S.A in the 1700s and was introduced to Western Europe soon after.

It is an annual flowering plant in the Malvaceae or mallow family which is cultivated for its edible green capsules rich in vitamins, carbohydrates, phosphorus, magnesium and potassium [5]. Economically, okra is sold to generate income for farmers [6]. Industrially, okra seed flour is used to fortify and improve the nutrient composition and functional properties of foods. Okra gum is used as a film coating agent for drug tablets [7] and is also used in the production of okra oil which is high in unsaturated fatty acids such as oleic and linoleic acids which may be suitable for use as biofuel [8, 9].

The major okra-producing countries in the world are India, Nigeria, Sudan, Mali, Pakistan, Cameroon, Ivory Coast, Ghana, and Benin. World okra production in 2018 was 9.87 million tons for a total cultivated area of 2.02 million hectares with India recording the highest production of 6 million tons and accounting for 62.0%. Cameroon produced 104,200 tons of okra in the same year [10].

The okra plant is propagated through seeds. The seed is one of the most crucial element in the livelihood of agricultural communities. It is the repository of the genetic potential of crop species and their varieties, resulting from continuous improvement and selection over time [11]. Seed systems are the ’vehicles’ through which farmers get good quality seeds for the crop varieties they need. An effective seed system has the potential to increase production quickly and economically [12]. Hence, more attention directed towards increasing seed yield with good quality for the successful production of okra is expected to drive the global market of okra [13, 14]. There are two seed production systems, the informal and formal seed production systems. The informal system comprises seeds derived from previous harvests, trade-by-barter and friends while the formal system is organized with a set of planned activities leading to the production of certified seeds. Both have a role to play in making seeds available for planting [11].

The growth and yield of okra depend upon various parameters including seed quality, agro-climatic and soil conditions, as well as agronomic practices [13]. Okra requires a long, warm and humid growing climate for better yield. It is sensitive to frost and extremely low temperatures [15], with an optimal temperature range of 21–30°C, maximum temperature of 35°C and a minimum of 18°C [15]. Okra requires well-drained sandy loam soil, with a pH between 5.8–6.5. Though drought-tolerant, okra requires a certain amount of water through irrigation. The adaptability of okra cultivars is greatly influenced by these biophysical conditions. That is why a given cultivar that is adaptable in agro-ecological zone A may not be adaptable in agro-ecological zone B. This explains the relationship between genes and the environment, which influences the phenotypic characteristics of crops.

It has been recognised that seeds more than any other farm input is the key to enhanced food production and increased income generation. Despite efforts to develop a national seed programme in Cameroon, the seed situation remains dismal. Hence, a proper seed production and distribution system is required to conveniently make available quality seeds for crop production.

In sub-Saharan Africa with Cameroon inclusive, the dominant technique used in producing okra seeds is the informal method. Moreover, okra has been considered a “minor” crop and very little attention has been paid to its improvement in research programmes in the past. Despite the presence of commercial okra hybrids in the market, farmers are reluctant to use them for various reasons.

The main objective of the study was to assess the informal seed production system of okra and evaluate the adaptability of seed produced from informal and formal systems under the agro-climatic and edaphic conditions of Buea. While the specific objectives were (i) to document the informal seed system of okra and (ii) to evaluate the adaptability of the okra seeds cultivated in Buea.

## Materials and methods

### Description of the study site

The study was carried out in Buea which is the headquarter of the South-West Region, in the Republic of Cameroon. Buea is located at 4°10’0” N of the Equator and 9°14’0” E of Greenwich Meridian and has an elevation of 870 m above sea level. Buea is geographically bounded to the North by the tropical rainforest at the foot of Mount Cameroon, to the South-west by Limbe, to the South-east by Tiko, to the East by Muyuka and to the West by Idenau. Buea falls within the humid forest agroecological zone, with mono-modal rainfall pattern. The average rainfall ranges from 3000–5000 mm annually, while the temperature ranges between 20–28°C. The area is composed mainly of volcanic rocks which range from massive basaltic lava flows around the upper slopes of Mount Cameroon to pyroclastic flows further down the slope [16]. The pH of the soil is between 5.58 and 5.74 [17]. The topography of Buea is composed of gentle slopes, with the sloping nature fairly stable throughout.

### Assessment of the informal seed system of okra in Buea

#### Reconnaissance survey and site selection

A reconnaissance survey was carried out to the sub-delegation of Agriculture in Buea to identify the major okra-producing areas in Buea municipality and the stakeholders involved in the okra sector. The areas identified were villages, which included; Small Soppo, Bwitingi, Molyko, Muea, Mile 16 (Bolifamba) and Wotutu.

#### Research design, field layout and application of treatments

A stratified random sampling method was used to select the informants needed to get information on seed production, cultivation, post-harvest management and marketing of okra in Buea. This sampling technique consists of partitioning the okra sector into sub-populations (Okra farmers, farmers shop owners, agricultural officers, marketers, consumers etc,)and randomly collecting data from each sub-population.

The field layout of the survey consisted of the various villages sampled which included Small Soppo, Bwitingi, Molyko, Muea, Mile 16, and Wotutu.

Test questionnaires were administered in the field and some corrections were effected before final administration to farmers. The different informants (stakeholders) sampled included; farmers, farmers’ shops, extension officers, marketers (retailers), consumers and some staff at the sub and regional delegation of agriculture and rural development. This triangulation helped to adequately collect relevant information on the informal seed system of okra in Buea.

### Evaluation of the adaptability of okra cultivars produced informally and formally

#### Land preparation, field layout, experimental design and application of treatments

**Fig 1:**
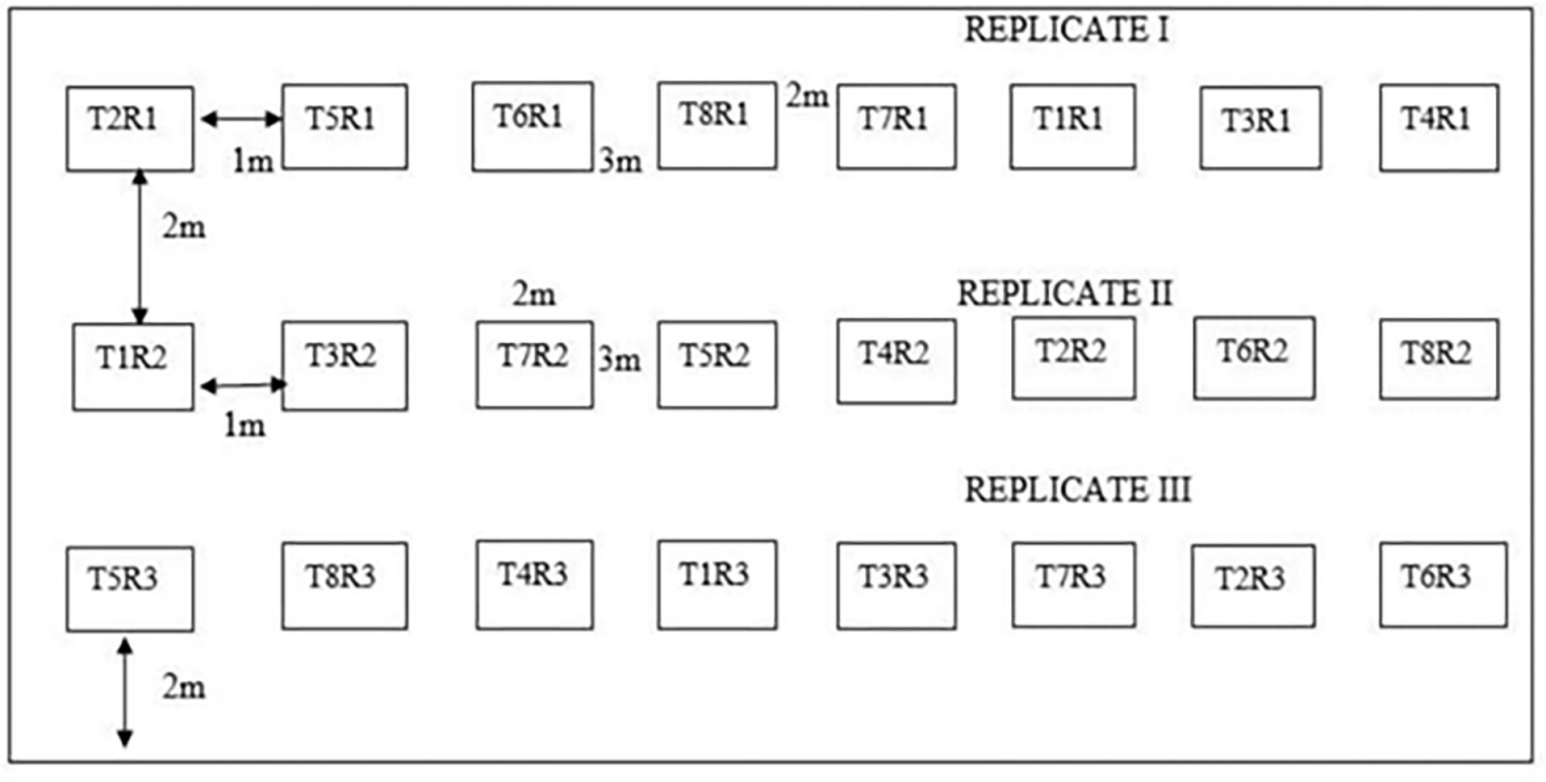
Field layout of the experiment.

A piece of land with a surface area of 420 m^2^ was cleared and gotten rid of stones and other unwanted materials with the help of a cutlass and rake. The experimental layout consisted of 3 replicates parallel to each other. Each replicate within the layout had 8 plots, making 24 experimental units in total. Plots of 2 m by 3 m (6 m^2^) each were tilled with the help of a hoe and raised to 30 cm height for easy root proliferation. A furrow of 1 m between each plot, 2 m between replicates and 2 m border around the experimental field were established. The experimental design used is a Randomised Complete Block Design (RCBD), with 8 treatments replicated 3 times (Fig 1). Randomisation of treatments on the experimental plots was done with the aid of a random number table. There were 6 intra-rows and 3 inter-rows giving a total of 18 stands per plot of 3 m x 2 m. Three (03) seeds were seeded per stand at a depth of 3 cm and later thinned to two plants per stand after two weeks, to give a total of 36 plants per plot. The seeds were sown at a planting distance of 50 cm by 75 cm, giving a plant density of 53,333 plants per hectare. The treatments for the experiment consisted of different cultivars of okra (Table 1). The seeds were purchased from local farmer shops in Buea and Douala, Cameroon.

**Table 1:** Okra cultivars used in this study and their sources.

**Codes**	**Treatment (Name of cultivar)**	**Source**
T1	Cafeier	Cameroon
T2	F1 Comet	Thailand
T3	Yellen	Thailand
T4	Longbec	Cameroon
T5	Gombo nain	Cameroon
T6	Clemson spineless	Thailand
T7	“Short” okra (landrace)	Cameroon
T8	F1 Kirikou	Cameroon

### Crop maintenance operations

#### Cultural control practices

*Weeding*. Weekly removal of weeds with a hoe was done from plots to avoid competition for nutrients, water and sunlight with the okra plants, as well as to remove potential hosts of pests.

*Thinning*. This is removing the less vigorous plant from a stand of three to reduce competition for nutrients and encourage seedling establishment and development.

*Replanting*. Replanting was done to replace seeds that did not germinate.

*Watering*. The plants relied on water from rain-fed and supplementary water, with the use of watering cans, during drought-prone conditions.

*Mulching*. This was done by placing dry and weeded grass around the root zone of crops to create a moist environment, which enhances the absorption of water and nutrients for the plants.

*Pruning*. Pruning was done with the use of a knife, to remove senesced and pests / disease infested leaves to avoid the build-up of pests and diseases.

*Earthing—up*. This is the mounting of top soil on the root zone of the crop, as the need arises to prevent root exposure and subsequent damage.

*Rogueing*. Diseased and genetically abnormal plants were removed and burnt to prevent further disease spread and other anomaly.

#### Chemical control practices

*Fertiliser application*. NPK (20:10:10) was applied at the rate of 10 g per stand under blanket application to promote vegetative growth and yield. This was done on the 3^rd^ and the 6^th^ week after planting (WAP), in split application with 5 g at each session.

*Pesticide application*. At the 5^th^ week after planting, the insecticide Inca with active ingredient Fipronil was applied, with 4 g dissolved in a 16 L knapsack sprayer. From the 8^th^ week after planting, the insecticide Acarius, with the active ingredient Abamectin was applied weekly at a rate of 50 ml/16L due to the build-up of whiteflies, ants and ladybird beetle. The fungicide Mancostar with active ingredient Mancozeb was applied weekly at the rate of 100 g/16L from the 8^th^ week after planting to control fungi.

### Data collection

#### The informal seed system of okra in Buea

Data collection instruments included the following: questionnaires, semi-guided discussion, focus group discussion, literature (secondary data), farm and market observation visits. One hundred (100) questionnaires were administered on a one-on-one basis to the farmers. Questions were properly explained before getting the answers from the respondents. In the course of the questionnaire administration, key informants were identified, who served as a backup for clarification as the need arose and were also invited to participate in the focus group discussion. The purpose of designing the questionnaire and administration was to find out; why the farmers plant okra (how beneficial it is to them), how they get their seeds (from the formal or informal seed sector) and why they choose to get them from there, how they produce their seeds (for those involved in seed production), the challenges faced in seed production (pests and diseases, lack of inputs, etc.), how they handle the seeds at postharvest, the challenges faced in storing / maintaining seed quality and how they market the seeds.

Semi-guided discussions with wholesalers and retailers of okra, as well as consumers and staff at the sub/regional delegation of agriculture and rural development in Buea, were held. Focus group discussions were held with key informants in the okra sector selected during the field survey. Secondary data from the literature review were used to complement knowledge already gathered from other data collection instruments. Field visits to okra farms as well as local markets were additional sources of data.

#### Evaluation of adaptability of okra cultivars produced informally and formally

*Germination percentage*. Data for germination was collected one week after seeding. This was done by getting the fraction of germinated seeds from the total and expressed in percentage.

*Vegetative growth parameters*. Data for vegetative growth was collected at 21, 36, 51 and 66 days after planting (DAP). Six stands in the middle of each plot were tagged, giving a total of 12 sampled plants, with a sampling intensity of 37.5%. Stands in the center were taken to minimize errors due to border effect. The following parameters were recorded:

**Plant height (cm):** This was done with the use of a measuring tape, from the base of the plant to the tip of the shoot.

**Number of leaves:** The number of fully opened leaves from the lower stem to the top of the plant was counted and recorded.

**Stem circumference (cm):** The stem circumference of tagged plants was recorded using a piece of thread round the stem of the plant and placing it on a meter rule to get the value.

**Leaf area (cm**^**2**^**):** The estimated leaf area of tagged plants was gotten with the use of a meter rule, positioned at three different points in the middle of the broadest leaf to the edge, to get an average and using the formula, estimated leaf area = Πr^2^.

**Number of branches:** Number of branches of tagged plants from the lower stem to the top of the plant was gotten by counting and recording on the data collection sheet.

*Common pests and diseases*. Data on pest and disease incidence and severity were collected at 51DAP, 66DAP and 81DAP.

**Incidence:** This is the percentage of diseased plants or parts in the sample or population of plants [18]. It was done by a visual counting of infected plants, divided by the total number of sampled plants and multiplied by 100.

Incidence = Number of infected plants X 100

Total number of sampled plants

**Severity:** This is the percentage of relevant host organs or tissues covered by symptoms or lesions or damaged by pest / disease. Severity results from the number and size of lesions.

Severity = Sum (Observed grade x number of plants in the same grade) X 100 [19]

Total number of plants evaluated x maximum grade on the scale

*Aphid and whitefly severity*. Assessment of severity by insect pests was done visually on a 0-5scale, where grade 0 = absence of aphids, grade 1 = scattered appearance of few aphids/whiteflies, grade 2 = few isolated colonies, grade 3 = several small colonies, grade 4 = large isolated colonies, grade 5 = large continuous colonies [20].

*Grasshopper severity*. Severity was estimated using a visual rating scale of 1–5 where 1 = 1–15 perforations on leaf (very mild damage), 2 = 16–30 perforations (mild damage), 3 = 31–45 perforations (moderately severe damage), 4 = 46–60 perforations (very severe damage), 5 = more than 60 perforations (extremely severe damage) [21].

*Wilt severity*. A visual scale of 0–4 was used to measure disease severity where 0 = plants with no symptoms, 1 = plant with no symptoms of wilt or yellowing, but with dark vascular bundles, 2 = plants with intensely darkened vascular bundles and with incipient wilt or yellowing of leaves, 3 = plants with severe wilt, associated with yellowing and premature leaf drop, 4 = dead plants [22].

*Powdery mildew severity*. The percentage of foliage disease severity was recorded by using the disease severity scale from 0–4 where 0 = no leaf lesions, 1 = 25% or less infected area of the leaf, 2 = 26–50% infected area of the leaf, 3 = 51–75% infected area of the leaf, 4 = 76–100% infected area of the leaf [23].

*Mosaic disease severity*. The assessment was carried out using a severity scale of 0–5 where 0 = no symptoms were observed, 1 = mild chlorosis, mottle or mosaic on leaves, 2 = mild chlorosis, mottle or mosaic without significant leaf distortion, 3 = moderate chlorosis, mottle or mosaic with leaf malformation, 4 = severe chlorosis, mottle or mosaic plus stunting or dwarfing of the whole plant, 5 = score 4 plus drying and leaf drop [24].

*Yield components and yield*. Data was collected at 66 DAP, 74 DAP and 82DAP for yield components and yield.

Number of okra capsules: The number of okra capsules was gotten by visually counting fully formed capsules and recording.

Length and width of okra capsules: The length and width were taken using a meter rule where:

Length of capsule/plant/treatment = Length of capsules harvested

Total number of capsules

Width of capsule/plant/treatment = Width of capsules harvested

Total number of capsules

**Fresh weight of okra capsule**: Fresh capsules were harvested and weighed using a scale balance.

**Yield:** This was gotten by multiplying the capsule weight per plant per treatment by the planting density.

### Data analysis

Data for the field survey was analysed by descriptive statistics using Microsoft Excel® 2016 version and SPSS version 25. A descriptive statistical method including percentage and frequency was used to summarize the ethnobotanical data collected through interviews and focus group discussions. Field experiment data were analysed by inferential statistics using a two-way ANOVA to test for significance and comparison of treatment means by Tukey’s Least Significance test at 5% probability level.

## Results

### The informal seed system of okra in Buea

#### Demographic information of respondents of the okra seed sector

Table 2 shows the personal information of the various respondents tending okra in Buea. Most okra farmers in Buea were women (60%) and the majority of farmers who cultivated okra fall within the age group of 40–49 years (42%). A great majority of the farmers were married (62%) and most of the respondents were primary school leavers (40%). In terms of farmers’ occupation, 70 respondents (70%) relied solely on farming for their livelihood.

**Table 2:** Personal information of respondents involved in informal seed system of okra.

S/N	Variable		Frequency	Percentage (%)
1	Gender	Male	40	40
		Female	60	60
2	Age	Less than (<)20 years	0	0
		20–29 years	20	20
		30–39 years	33	33
		40–49 years	42	42
		Above (>) 50 years	5	5
3	Marital status	Single	28	28
		Married	62	62
		Divorced	4	4
		Widow/widower	6	6
4	Level of education	Primary education	40	40
		Secondary education	17	17
		High school	12	12
		University education	5	5
		Vocational training	26	26
5	Occupation	Farming	70	70
		Off-farm activities	30	30

### Seed production and okra cultivation

Most farmers obtained planting materials from mass selection (choosing from the healthiest capsules of the previous harvests), representing 42 respondents (42%) relative to 20 farmers (20%) using certified seeds bought from farmers’ shops, 19 (19%) using seeds bought from the market, 12 (12%) using seeds from family and 7(7%) using seeds gotten from friends (Table 3). During cultivation, the majority of the farmers 78 (78%) adopted a mixed cropping system while 22 respondents (22%) adopted monoculture. In terms of the performance of the landrace and certified seeds, 74 (74%) of the respondents reported the landrace to perform better than the certified seeds. The plot sizes used in okra cultivation in Buea ranged between 25–20,000 m^2^ and majority of the farmers either used personal land (inherited or purchased) while others used rented land. Experience in okra cultivation among the respondent ranged from one to fifty years.

**Table 3:** Seed production by respondents involved in the informal seed system of okra.

S/N	Variable		Frequency	Percentage (%)
1	Source of planting material	Previous harvest	42	42
		Family	12	12
		Friends	7	7
		Market	19	19
		Farmer shop	20	20
2	Cropping system used	Mono cropping	22	22
		Mixed cropping	78	78
3	Highest yielding cultivar	Landrace	74	74
		Hybrid	26	26

Two cultivated landraces were identified, that is, the “3 months” (annual) landrace and the “6 months” (biennial) landrace.

### Facilities used for okra preservation and storage in Buea

Farmers use different preservatives to improve the shelf-life of okra seeds during storage. Fifty-eight of the respondents (58%) used wood ash as a form of preservative, while 27 respondents (27%) reported the use of assorted preservatives (excluding chemical options) before storage. Just 15 respondents (15%) used chemicals like Mocap 10G as a preservative for okra seeds.

Farmers in Buea used different media for the storage of okra seeds. While 70 (70%) of the respondents used plastic containers for okra seed storage, 16 (16%) of them allowed the seeds in the capsules and 14 (14%) used cloth bags as their storage medium. In terms of the cause of seed deterioration during storage, 69 (69%) of the respondents attest that weevils were the major insect pest. Concerning challenges during storage,76 (76%) of the respondents opined that attack by insect pests were the most prominent compared to fungal and animal pests as shown in Table 4.

**Table 4:** Okra seed preservation and storage.

S/N	Variable		Frequency	Percentage (%)
1.	Preservation of seeds	Spraying with wood ash	58	58
		Use of a chemical	15	15
		Use of a plant extract	0	0
		No use of preservative	27	27
2.	Seed storage	Containers	70	70
		Bags	14	14
		Okra capsules	16	16
3.	Insect pests	Ants	2	2
		Beetles	7	7
		Mites	12	12
		Weevils	69	69
		None	10	10
4.	Seed storage challenges	Attack by insect pests	76	76
		Attack by fungal diseases	3	3
		Attack by animal pests	5	5
		Seeds dry out and can no longer germinate	6	6
		None	10	10

### Evaluation of adaptability of okra cultivars produced informally and formally

#### Germination percentage of okra cultivars

Germination percentage is an indicator of seedling vigour and plays a role in seed establishment. While F1 Comet recorded the highest germination percentage (93.46%), the cultivar with the least germination percentage was Nain with 33.53% (Table 5).

**Table 5:** Germination percentage of okra cultivars.

Cultivar	Cafeier	F1 Comet	Yellen	Longbec	Nain	Clemson Spineless	Landrace	Kirikou
Germination (in %)	79.69	93.46	73.1	61.11	33.53	68.4	63.83	88.83

### Vegetative growth parameters of okra cultivars

*Plant height*. For the plant height, F1 Comet recorded the highest value (64.36 cm) which was consistently significant across the different sampling periods (Table 6). At 21 days after planting (DAP), the height of F1 Comet (8.43 cm) was not significantly different from Clemson spineless, the Landrace, Kirikou and Yellen but was significantly different from Longbec, Nain and Cafeier.

**Table 6:** Plant height of okra cultivar.

Cultivar	21 DAP	36 DAP	51 DAP	66 DAP
**Cafeier**	2.2±0.1c	3.8±0.6b	12.5±1.5b	20.5±2.2cd
**Clemson**	6.8±1.4ab	12.9±3.0ab	28.6±6.0ab	38.6±2.8bc
**F1 Comet**	8.4±0.7a	15.2±2.4a	42.5±6.7a	64.4±2.5a
**Kirikou**	5.5±0.9abc	11.1±2.6ab	29.4±7.1ab	37.1±9.0bcd
**Landrace**	5.7±1.4abc	11.1±3.1ab	32.2±8.5ab	42.3±7.2b
**Longbec**	3.6±0.8bc	7.3±0.3ab	19.0±0.3ab	23.6±0.1bcd
**Nain**	2.8±0.2bc	5.0±0.2b	10.9±1.1b	16.3±0.9d
**Yellen**	5.0±0.2abc	8.8±0.7ab	22.1±1.5ab	32.9±0.6bcd

Values with the same letter in a column are not significantly different from each other under Tukey test at a 5% probability level.

At 36 DAP, the height of the F1 Comet cultivar (15.16 cm) was also not significantly different from Clemson spineless, the Landrace, Kirikou, Yellen, Longbec but was significantly taller than Nain and Cafeier.

The height of F1 Comet (42.50 cm) was not significantly different from the Landrace, Kirikou, Clemson spineless, Yellen, Longbec, but was significantly different from Cafeier and Nain at 51DAP.

F1 Comet was significantly taller (64.36 cm) than the other cultivars at 66 DAP. The Landrace was second in terms of height (42.25 cm), which was not significantly different from Clemson spineless, Kirikou, Yellen, Longbec, but significantly different from Cafeier and Nain.

*Stem circumference*. All the okra cultivars recorded increasing values for stem circumference across the different weeks of measurement (Table 7). Cultivars did not record any significant results at the 21, 36 and 51 DAP respectively. At 66 DAP, Kirikou recorded the highest value (5.00 cm) for stem circumference, which was significantly different from Longbec, but not significantly different from the other cultivars examined.

**Table 7:** Stem circumference of okra cultivars.

Cultivar	21 DAP	36 DAP	51 DAP	66 DAP
**Cafeier**	1.5±0.1a	1.9±0.1a	3.3±0.2a	4.1±0.2ab
**Clemson**	1.3±0.0a	2.2±0.2a	3.7±0.4a	4.5±0.4ab
**F1 Comet**	1.6±0.1a	2.5±0.3a	3.9±0.2a	4.7±0.2ab
**Kirikou**	1.4±0.1a	2.5±0.3a	4.1±0.5a	5.0±0.4a
**Landrace**	1.5±0.2a	2.7±0.6a	3.7±0.7a	4.4±0.7ab
**Longbec**	1.3±0.1a	2.3±0.1a	2.7±0.1a	3.1±0.0b
**Nain**	1.3±0.0a	1.8±0.0a	2.9±0.1a	3.3±0.1ab
**Yellen**	1.6±0.0a	2.3±0.1a	3.8±0.3a	4.6±0.3ab

Values with the same letter in a column are not significantly different from each other under Tukey test at a 5% probability level.

*Number of leaves*. Okra cultivars showed robust growth throughout the sampling period for the number of leaves (Table 8). At 21 DAP, the Landrace and F1 Comet recorded the highest values (4.67), which was not significantly different from the other cultivars but was significantly different from Longbec. Cultivars did not record any significant results at 36 and 51DAP respectively. At 66DAP, Yellen recorded the highest value (13.42), which was not significantly different from the other cultivars but was significantly different from Clemson spineless.

**Table 8:** Number of leaves of okra cultivars.

Cultivar	21 DAP	36 DAP	51 DAP	66 DAP
**Cafeier**	4±0.1ab	6±0.2a	8±0.3a	10±1.0ab
**Clemson**	4±0.1ab	7±0.2a	7±0.5a	7±1.4b
**F1 Comet**	5±0.3a	7±0.3a	10±1.3a	13±0.9a
**Kirikou**	4±0.4ab	6±0.5a	10± 1.8a	11±1.8ab
**Landrace**	5±0.3a	6±0.6a	8±1.3a	11±1.0ab
**Longbec**	3±0.3b	5±0.1a	8±0.5a	10±0.6ab
**Nain**	3±0.4ab	5±0.3a	8±0.5a	10±0.6ab
**Yellen**	4±0.1ab	6±0.2a	9±1.2a	13±0.9a

Values with the same letter in a column are not significantly different from each other under Tukey test at a 5% probability level.

*Leaf area*. Analysis of variance revealed that okra cultivars recorded significant results at 21, 36 and 51 DAP respectively (Table 9). At 21 DAP, F1 Comet recorded the highest leaf area (30.93 cm^2^), which was not significantly different from Kirikou, Yellen, Landrace, and Clemson spineless but significantly different from Longbec, Nain and Cafeier. At 36 DAP, F1 Comet recorded the highest leaf area (181.8 cm^2^), which was not significantly different from the Landrace and Kirikou, but was significantly higher than Clemson spineless, Yellen, Longbec, Nain and Cafeier. At 51 DAP, Kirikou recorded the highest leaf area (447.7 cm^2^) which was not significantly different from Yellen but was significantly different from F1 Comet, Clemson spineless, Landrace, Cafeier, Longbec and Nain. At 66 DAP, Yellen recorded the highest value for leaf area (771.4 cm^2^), which was significantly different from Kirikou, F1 Comet, Clemson spineless, Landrace, Longbec, Nain and Cafeier.

**Table 9:** Leaf area of okra cultivars (in cm^2^).

Cultivar	21 DAP	36 DAP	51 DAP	66 DAP
**Cafeier**	7.2±1.2c	34.1±4.2e	163.1±12.0d	232.4±14.8d
**Clemson**	18.5±3.8abc	136.7±6.5bc	321.9±11.7c	445.1±19.7c
**F1 Comet**	30.9±3.8a	181.8±10.1a	342.1±12.2bc	481.7±23.7c
**Kirikou**	25.4±2.3ab	153.7±9.0ab	447.7±26.1a	668.7±26.2b
**Landrace**	20.9±2.5ab	153.9±8.1ab	303.1±18.5c	441.5±17.2c
**Longbec**	17.1±1.7bc	115.9±4.6cd	161.2±9.0d	274.3±23.5d
**Nain**	16.1±2.3bc	85.1±11.0d	127.0±12.1d	233.1±14.8d
**Yellen**	23.9±2.3ab	121.7±4.6bcd	420.1±19.7ab	771.4±21.1a

Values with the same letter in a column are not significantly different from each other under Tukey test at a 5% probability level.

*Number of branches*. At 36DAP, Kirikou and Yellen recorded the highest value (2.47) for the number of branches per plant, which was not significantly different from F1 Comet and Cafeier, but significantly out-numbered those of other cultivars (Table 10). At 51DAP, Kirikou also recorded the highest value for the number of branches (3.97), which was not significantly different from Yellen and Cafeier, but was significantly different from the other cultivars. At 66DAP, Yellen recorded the highest value (5.33), which was not significantly different from Kirikou and Cafeier, but was significantly higher than the other cultivars.

**Table 10:** Number of branches of okra cultivars.

Cultivar	36 DAP	51 DAP	66 DAP
**Cafeier**	2±0.3ab	3±0.5ab	5±0.2a
**Clemson**	1±0.2cd	2±0.1c	3±0.1c
**F1 Comet**	2±0.1ab	3±0.1bc	3±0.1bc
**Kirikou**	3±0.0a	4±0.1a	5±0.2a
**Landrace**	2±0.1bc	3±0.3bc	4±0.4b
**Longbec**	1±0.1d	2±0.1c	3±0.3bc
**Nain**	1±0.2bcd	2±0.1c	3±0.1bc
**Yellen**	3±0.0a	4±0.2a	6±0.1a

Values with the same letter in a column are not significantly different from each other under Tukey test at a 5% probability level.

### Incidence and severity of common pests and diseases on okra cultivars in Buea

*Incidence of pests and diseases on okra cultivars*. Okra cultivars recorded differences with respect to the incidence of pests and diseases across weeks. At 51DAP, Clemson spineless recorded the highest incidence (80.87%), which was significantly different from the other cultivars (Table 11). This was closely followed by Longbec (50.67%), which was also significantly different from the other cultivars and lastly, F1 Comet recorded the third-highest value (8.17%), which was not significantly different from the other cultivars. At 66 and 81 DAP, Clemson spineless recorded the highest incidence (81.27% and 89.90% respectively), which were significantly different from the other cultivars.

**Table 11:** Pest and disease incidence on okra cultivars.

Cultivar	51DAP	66DAP	81DAP
Cafeier	0.00 ±0.00c	0.00 ±0.00d	0.00 ±0.0d
Clemson	80.87 ±5.42a	81.27 ±5.72a	89.90 ±4.14a
F1 Comet	8.17 ±0.52c	16.47 ±2.23c	25.00 ±2.89c
Kirikou	0.00 ±0.00c	0.00 ±0.00d	0.00 ±0.00d
Landrace	0.00 ±0.00c	0.00 ±0.00d	0.00 ±0.00d
Longbec	50.67 ±2.33b	58.20 ±3.55b	67.23 ±4.34b
Nain	0.00 ±0.00c	0.00 ±0.00d	0.00 ±0.00d
Yellen	0.00 ±0.00c	0.00±0.00d	0.00 ±0.00d

Means that do not share the same letter in a column are significantly different under a Tukey test at a 5% probability.

*Severity of common pests on okra cultivars in Buea*: *The severity of aphids and whiteflies on okra cultivars*. The severity of infestation of okra cultivars to aphids and whiteflies varied among cultivars within and across the sampling periods (Table 12). At 51DAP, Clemson spineless recorded the highest value (3.33), which was not significantly different from Longbec, Yellen and F1 Comet but was significantly different from the other cultivars. At 66DAP, Clemson spineless still recorded the highest value (3.67), which was not significantly different from Longbec, Yellen and Kirikou, but was significantly different from the other cultivars. At 81DAP, Clemson spineless again recorded the highest value (3.67), which was not significantly different from Longbec and F1 Comet, but was significantly different from the other cultivars.

**Table 12:** Severity of Aphid and whitefly on okra cultivars.

Cultivar	51DAP	66DAP	81DAP
Cafeier	1.0±0.0b	1.3±0.3b	1.1±0.1b
Clemson	3.3±0.3a	3.7±0.9a	3.7±0.9a
F1 Comet	1.7±0.3ab	1.5±0.3b	1.8±0.2ab
Kirikou	1.3±0.3b	1.7±0.3ab	1.3±0.3b
Landrace	1.1±0.1b	1.3±0.3b	1.0±0.0b
Longbec	2.7±0.9ab	2.7±0.3ab	2.3±0.3ab
Nain	1.0±0.0b	1.3±0.3b	1.5±0.3b
Yellen	1.7±0.3ab	1.7±0.3ab	1.3±0.3b

Values with the same letter in a column are not significantly different from each other under Tukey test at a 5% probability level.

*The severity of grasshoppers on okra cultivars*. At 51, 66 and 81 DAP, Clemson spineless recorded the highest severity (3.67,4.17 and 3.50 respectively) for grasshoppers which significantly out-numbered that of the other cultivars (Table 13).

**Table 13:** Severity of grasshoppers on okra cultivars.

Cultivar	51DAP	66DAP	81DAP
Cafeier	1.2±0.2b	1.4±0.4b	1.3±0.3b
Clemson	3.7±0.3a	4.2±0.2a	3.5±0.3a
F1 Comet	1.3±0.3b	1.5±0.3b	1.2±0.2b
Kirikou	1.5±0.3b	1.3±0.3b	1.5±0.3b
Landrace	1.2±0.2b	1.5±0.5b	1.2±0.2b
Longbec	2.0±0.3b	1.3±0.3b	1.7±0.4b
Nain	1.5±0.3b	1.5±0.5b	1.2±0.2b
Yellen	1.4±0.3b	1.8±0.4b	1.7±0.4b

Values with the same letter in a column are not significantly different from each other under Tukey test at a 5% probability level.

*The severity of wilt disease infestation on okra cultivars*. Table 14 presents the results of the severity of infestation of wilt disease on okra cultivars in Buea. Apart from Longbec and F1 Comet that were not significantly different from Clemson spineless that registered the highest severity of wilt disease infestation of 4.33 at 51 and 81 DAP respectively, it (Clemson spineless) was significantly different from the other cultivars and all the cultivars at 66 DAP with a peak infestation value of 4.67.

**Table 14:** Severity of wilt on okra cultivars.

Cultivar	51DAP	66DAP	81DAP
Cafeier	1.7±0.3bc	1.3±0.3b	1.7±0.3b
Clemson	4.3±0.3a	4.7±0.3a	4.3±0.7a
F1 Comet	3.0±0.6abc	2.0±0.0b	1.5±0.3b
Kirikou	1.8±0.3bc	1.7±0.3b	1.3±0.3b
Landrace	1.3±0.3c	1.3±0.3b	1.2±0.2b
Longbec	3.3±0.3ab	2.7±0.3b	3.0±0.6ab
Nain	1.7±0.3bc	1.7±0.3b	1.7±0.7b
Yellen	1.3±0.3c	1.3±0.3b	1.7±0.3b

Values with the same letter in a column are not significantly different from each other under Tukey test at a 5% probability level.

*The severity of powdery mildew infestation on okra cultivars*. The results of the severity of powdery mildew infestation on okra cultivars are presented in Table 15. Clemson spineless registered the greatest severity of infestation to powdery mildew at all sampling dates (1.30, 1.63 and 2.00 at 51, 66 and 81 DAP respectively), which were significantly different from the values recorded by the other cultivars except for Longbec at 66 and 81 DAP.

**Table 15:** Severity of powdery mildew on okra cultivars.

Cultivar	51DAP	66DAP	81DAP
Cafeier	0.0±0.0b	0.0±0.0b	0.0±0.0b
Clemson	1.3±0.3a	1.6±0.3a	2.0±0.3a
F1 Comet	0.0±0.0b	0.0±0.0b	0.0±0.0b
Kirikou	0.0±0.0b	0.0±0.0b	0.0±0.0b
Landrace	0.0±0.0b	0.0±0.0b	0.0±0.0b
Longbec	0.0±0.0b	1.2±0.2a	1.5±0.3a
Nain	0.0±0.0b	0.0±0.0b	0.0±0.0b
Yellen	0.0±0.0b	0.0±0.0b	0.0±0.0b

Values with the same letter in a column are not significantly different from each other under Tukey test at a 5% probability level.

*The severity of mosaic infestation on okra cultivars*. At 51DAP, Clemson spineless recorded the highest severity of infestation (2.83), which was significantly different from the other cultivars. At 66DAP, Clemson spineless still recorded the highest severity of infestation (2.67), which was not significantly different from the other cultivars. Likewise, at 81DAP, Clemson spineless again recorded the highest severity of infestation (4.50), which was significantly different from the other cultivars, as shown in Table 16.

**Table 16:** Severity of mosaic disease on okra cultivars.

Cultivar	51DAP	66DAP	81DAP
Cafeier	1.2±0.2b	1.5±0.3a	1.3±0.3bc
Clemson	2.8±0.4a	2.7±0.3a	4.5±0.3a
F1 Comet	1.2±0.2b	2.5±1.3a	1.3±0.3bc
Kirikou	1.5±0.3b	1.5±0.3a	1.2±0.2c
Landrace	1.2±0.3b	1.3±0.2a	1.3±0.2bc
Longbec	1.3±0.3b	1.5±0.3a	2.7±0.3b
Nain	1.2±0.2b	1.3±0.3a	1.3±0.3bc
Yellen	1.2±0.2b	1.5±0.3a	1.7±0.3bc

Values with the same letter in a column are not significantly different from each other under Tukey test at a 5% probability level.

#### Yield components of okra cultivars grown in Buea

**Fig 2:**
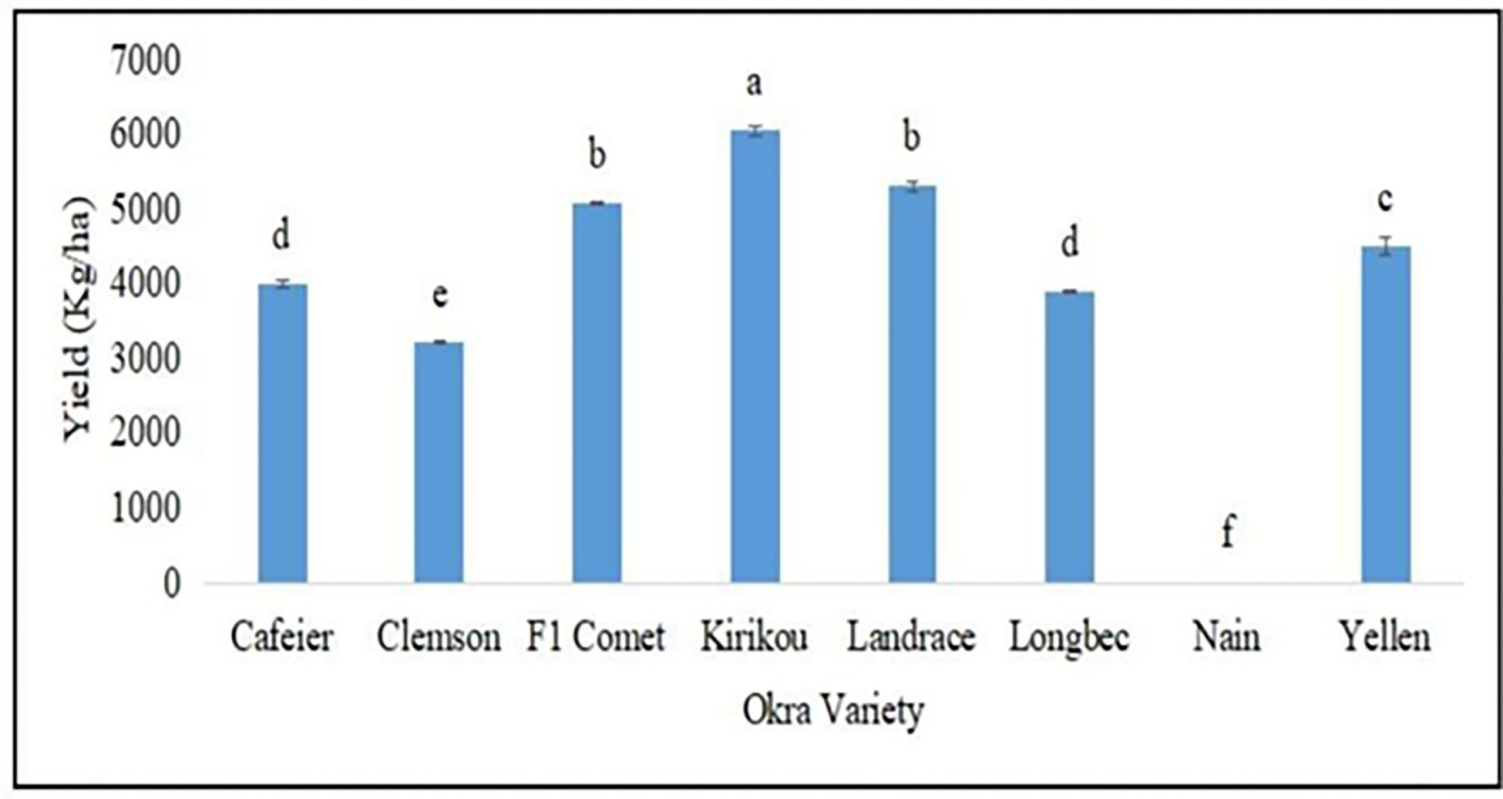
Yield of okra cultivars planted in Buea, Cameroon (kg/ha).

Concerning the yield components of okra cultivars, F1 Comet recorded the highest value for capsule length (19.72 cm), which was significantly different from the other cultivars. The Landrace recorded the highest value for capsule width (6.19 cm), which was also significantly different from the other cultivars. Kirikou recorded the highest value for the number of capsules (14.03), which was not significantly different from Yellen, Cafeier, Landrace and F1 Comet, but was significantly different from the other cultivars. With respect to capsule weight and yield, Kirikou again recorded the highest values (0.11 kg/plant/treatment and 6.05t/ha) respectively, which was significantly different from the other cultivars (Table 17 and Fig 2).

**Table 17:** Yield components of okra cultivars cultivated.

Cultivar	Capsule Length (cm)	Capsule Width (cm)	Number of capsules/Plant	Capsule weight (kg/plant/treatment)
Cafeier	4.99 ± 0.61e	3.00 ± 0.0c	11.61 ± 0.85a	0.07 ±0.0029cd
Clemson	6.23 ± 0.14d	4.06 ± 0.04b	5.92 ± 0.08b	0.06 ±0.0029e
F1 Comet	19.72 ± 0.15a	3.04 ± 0.02c	11.17 ± 1.09a	0.09 ±0.0021b
Kirikou	6.76 ± 0.21cd	4.28 ± 0.17b	14.03 ± 0.48a	0.11 ±0.0017a
Landrace	12.36 ± 0.15b	6.19 ± 0.16a	11.56 ± 1.18a	0.10±0.0017b
Longbec	7.54 ± 0.52c	2.17 ± 0.16d	3.66± 0.26b	0.07 ±0.0020d
Nain	0.00±0.00f	0.00±0.00e	0.00±0.00c	0.00 ±0.0000f
Yellen	5.97 ± 0.05de	3.82 ± 0.03b	12.31 ± 0.84a	0.08 ±0.0023c

Means that do not share the same letter in a column are significantly different under Tukey test at a 5% probability.

**Fig 3:**
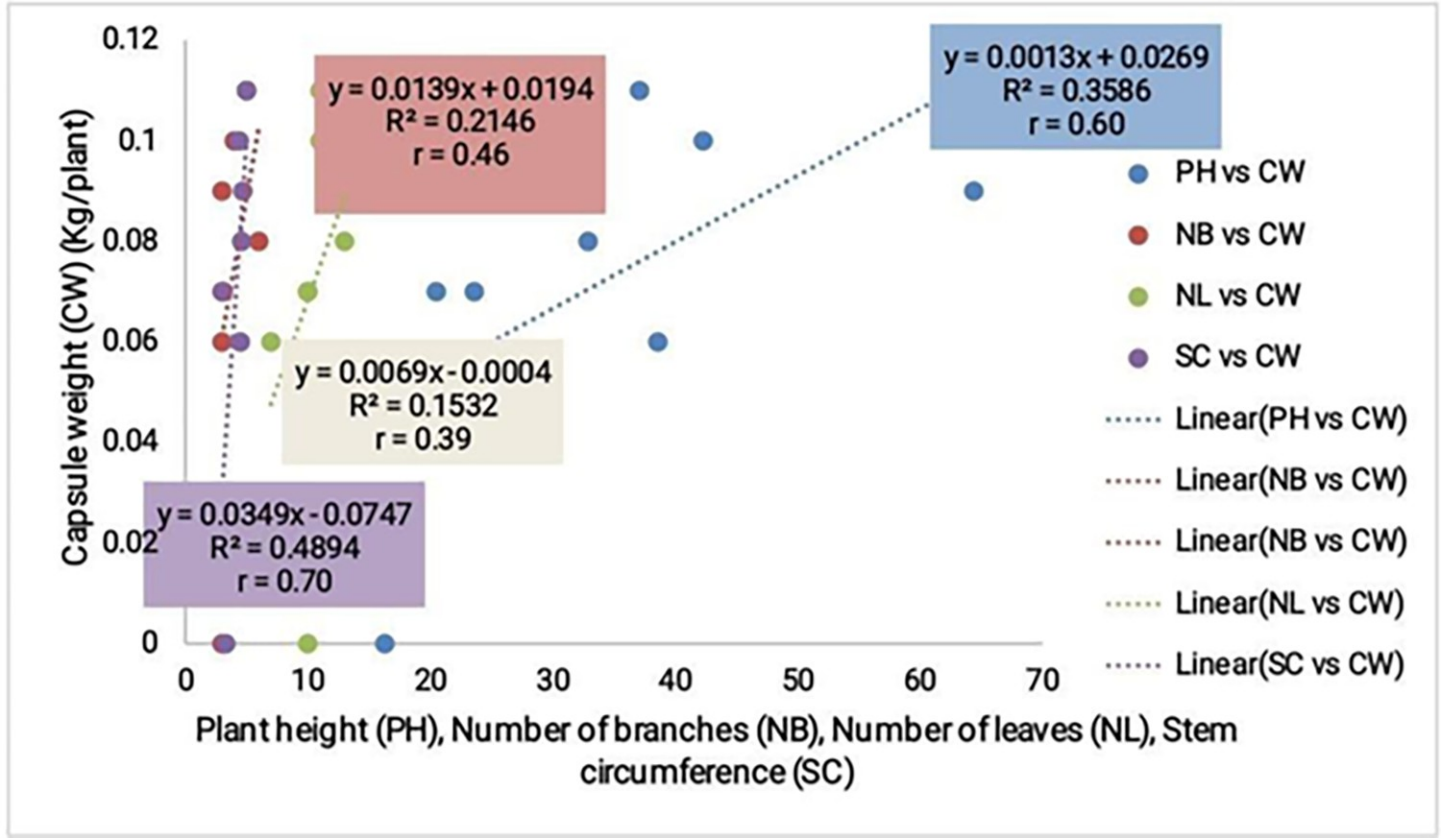
Correlation of vegetative growth parameters with okra yield.

**Fig 4:**
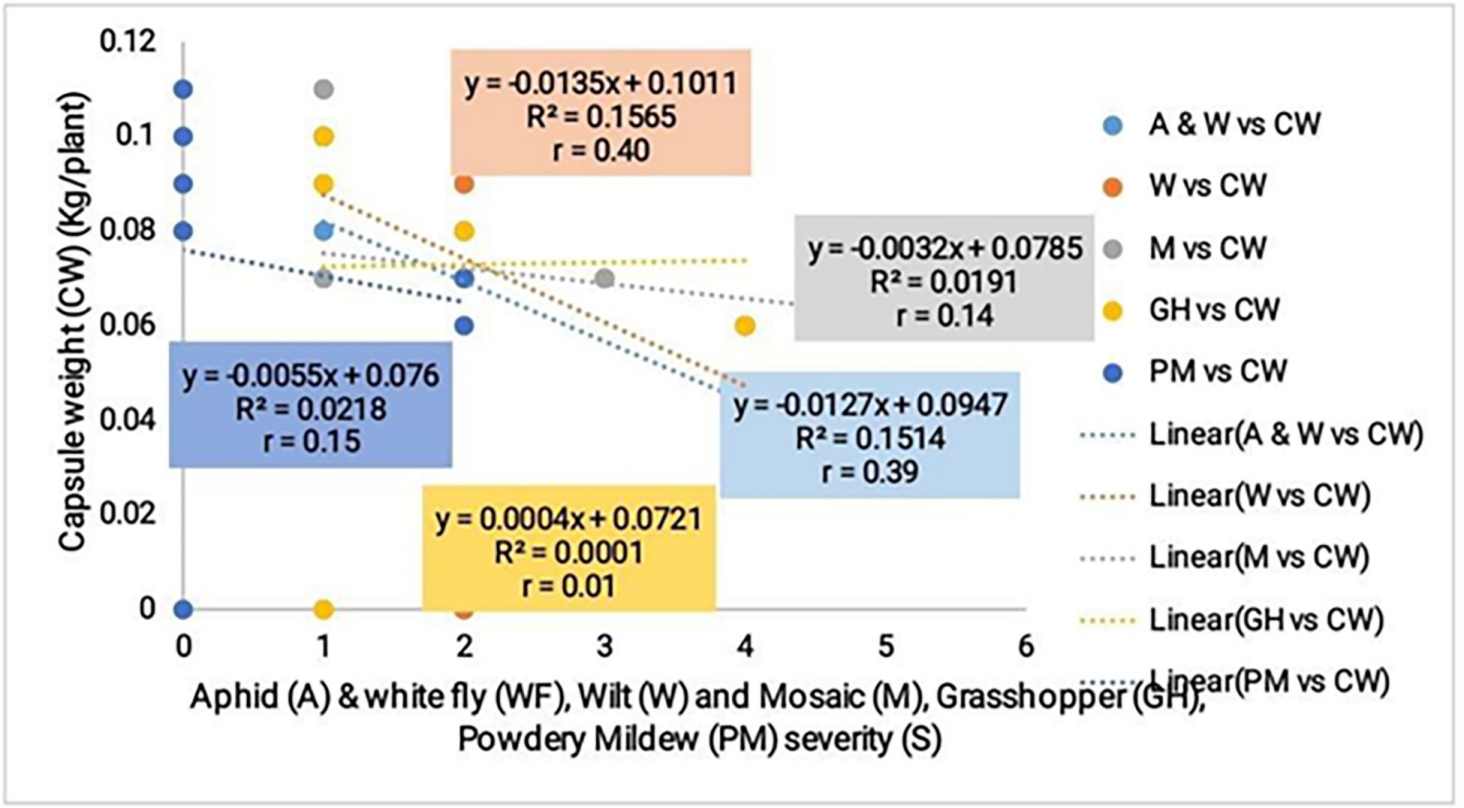
Correlation of pest and disease severity with okra yield.

*Correlation of vegetative growth parameters and pest / disease severity with okra yield*. A positive correlation was noticed for all vegetative growth parameters compared with the yield with stronger correlation values for plant height (r = 0.6) and stem circumference (r = 0.7) (Fig 3). While pest and disease was inversely proportional to the yield and registered very weak correlation values (Fig 4).

## Discussion

### The informal seed system of okra in Buea

Most of the okra farmers involved in informal seed production and cultivation were women of the middle age group (40–49 years) who are married and had at least a primary school education. This conforms with research carried out by Tata Ngome *et al*. [25] in 3 towns of Cameroon (Bafoussam, Buea and Ebolowa) and reported that a greater percentage of vegetable farmers in Buea and Ebolowa were women while those in Bafoussam were men. On the other hand, Farinde *et al*. [26], showed that the males in Egbedore Local Government Area, Osun State of Nigeria, were the ones who cultivated okra and the women participated at the level of processing, preservation and marketing. The disparity in gender with more female farmers can have an influence on the cropping system used for seed / okra production, consumption and marketing prerogatives. This study also shows that it was the middle-age group who were mostly concerned with okra farming as the youths showed little interest, which ties with studies by Farinde *et al*. [26], who pointed out that only 4% of respondents in their study were youths. The system of seed production and okra cultivation are the informal seed system and small-scale production system respectively, characterize with insufficient capital, low level of technology and intensive labour, thus making it not friendly and appetizing for the youths.

Farmers mostly used mass selection to produce planting materials. This conforms to a study carried out by Phippen [27] on the evaluation of nine okra varieties for seed production where he concluded that even hybrid okra varieties still have a tremendous amount of genetic diversity between them to support a breeding program for increasing seed yield. Ahiakpa *et al*. [28] also presented similar findings and said that traditional farmers often engage in selection for different purposes, facilitating its diversification into a vast number of landraces adapted to various agroecological systems. Massucato *et al*. [29] in a study conducted on 30 accessions of okra in Brazil, noted that small farmers usually store some of the seeds to sow during the next cropping season, generation after generation. The dominant cropping system for both seed production and okra cultivation was mixed cropping, followed by monoculture. The mixed cropping system used by okra farmers has also been highlighted previously by Tata Ngome *et al*. [25]. Farmers attributed the landrace to favourably compete with the commercial okra hybrids in terms of vigour, pest and disease resistance, as well as yield. In a related study by Tata Ngome *et al*. [25], it was noticed that while farmers in Buea and Ebolowa used seeds from mass selection, on the contrary, farmers in Bafoussam used commercial hybrids as their planting materials. The informal system of okra seed production essentially by mass selection is questionable with respect to seed quality due to the absence of temporary and spatial isolation conditions to avoid the introgression of foreign genes which could result in ’contamination’ of the progeny’s genetic integrity and the expression of phenotypic traits. The situation is checked by the crops autogamous nature and coupled to the fact that the percentage of allogamy is greatly reduced, since dehiscence of the anthers / pollination at anthesis has a restricted time interval.

The cultivar preferred by most of the farmers according to the results was the landrace (*Abelmoschus caillei* (A. Chev.) Stevels). This is similar to findings by Farinde *et al*. [26], who found that farmers preferred the landrace compared to the commercial hybrids for their planting materials. These landraces may have relevant traits for vigour, resistance, tolerance and improved yield, coupled with the autogamous nature of the crop [30, 31].

Farmers used indigenous postharvest handling methods, processing techniques, preservatives and storage media to improve the viability and shelf-life of okra seeds [14]. Amongst the preservatives and storage media used, wood ash and plastic containers were dominantly used by farmers to preserve and store seed, respectively, in accordance with [25]. Meanwhile, wood ash is relevant in absorbing water and rendering the storage medium constantly arid, plastic containers are good in providing an air-tight environment, which goes a long way in reducing the deterioration rate of the seeds. Farmers were faced with limitations in terms of the utilization of preservatives and storage technology for okra seeds and common postharvest pests that infested okra seeds were insects.

### Evaluation of the adaptability of okra cultivars

#### Seed germination and vegetative growth of okra cultivars

Seed germination constitutes a major parameter of vigour and adaptability. Meanwhile, orthodox seeds have a better shelf life, viability and germination percentage, this is the reverse for recalcitrant seeds. Seed quality attributes and environmental factors also greatly influences the breaking down of seed dormancy and germination percentage.

For plant height, F1 Comet recorded significant results within cultivars and across periods of data collection. Other cultivars which recorded better results for plant height in descending order were the Landrace, Clemson spineless, Kirikou, Yellen, Longbec, Cafeier and Nain. Plant height is important in sunlight interception, especially in interspecific competition conditions, since it exposes the leaves of heliophytic plants to sunlight radiation for food production. The variation in plant height among the different okra cultivars may also be due to the fact that some varieties are early maturing (such as Clemson spineless, F1 Comet), medium maturing (Longbec, Kirikou, Landrace, Yellen) and late maturing (such as Cafeier) and thus, have different growth rates. Farooq *et al*. [32] also opined that there were significant variations in growth habits for all the five varieties of okra under experimentation (Penta green, Pusa sawani, Local cultivar, Pusa green and Clemson spineless) in India. The result also coincided with the findings of Omonhinmin and Osawaru [33], who stated that different okra cultivars showed varied plant heights at different growth stages. Nsimi *et al*. [34] corroborated that the highest plant height was registered from a local okra cultivar while an imported cultivar (Clemson spineless) had the least height.

For stem circumference, Kirikou recorded the most significant results within cultivars and across the data collection schedule while the other cultivars with better results for stem circumferemce in descending order were F1 Comet, Yellen, Clemson spineless, the Landrace, Cafeier, Nain and Longbec. The stem is relevant in maintaining an upright position, especially for plants with erect growth habits. It also acts as a pathway for the transportation and translocation of water/nutrients and food substances respectively. The larger the stem girth, together with the type of tissues, the lesser the possibility of lodging especially during stormy climatic conditions. This is in tandem with studies by Temam *et al*. [35] who reported differences in stem girths of various okra cultivars ranging from 14.5–30.5 cm in Ethiopia. These results contradict the findings of Azeem *et al*., [36] who reported an insignificant difference in stem girth among the various okra cultivars under his study.

In terms of the number of leaves, the cultivar that produced the most significant results was F1 Comet. While leaves are important for sunlight interception, the more the number of leaves on a plant, the greater the possibility for the plant to produce more food. The increased production in the number of leaves, across the developmental stages of the crop, may be attributed to the vigour of the cultivar and increased absorption of nutrients which resulted in increased synthesis of photosynthates. Similar findings by Saleem *et al*. [37] revealed differences in the number of leaves recorded for okra cultivars under test due to high phenotypic and genotypic variance. Alam and Hossain [38] also found significant differences in number of leaves for the different accessions of okra tested. Chadha *et al*., [39] documented significant differences in the number of leaves and observed that okra cultivars with more leaves per plant produced higher fruit yields.

With regard to the leaf area, the cultivar that recorded the most significant results was Yellen. The other cultivars in descending order of importance were Kirikou, F1 Comet, Clemson spineless, the Landrace, Longbec, Cafeier and Nain. Broader leaves have the tendency of intercepting more sunlight radiation and thus, the possibility of producing more food. Leaf radius is directly proportional to the leaf area. So, a larger leaf radius means a larger leaf area [37].

For number of branches, the cultivar with an outstanding result is Yellen and was closely followed by Kirikou, Cafeier, the Landrace, Nain, F1 Comet, Longbec and Clemson spineless. Under favourable growing conditions, plants with more branches will contain more leaves and have greater chances of photo assimilation. Branches are important as they are the sites for leaf attachment and growth and add some support to the plant. The discrepancy in the number of branches per cultivar might be due to their genetic make-up and environmental factors. Similar findings by Alam and Hossain [38] showed significant variation in the number of branches of okra accessions under study. Also, they stated that little differences were found between the genotypic and phenotypic variance as well as between the genotypic and phenotypic coefficients of variation, indicating low environmental influence.

Cultivar specificity in performance with respect to germination percentage and vegetative growth parameters are important predictions of vigorous growth and adaptability in a given environment.

#### Incidence and severity of pests and diseases on okra cultivars

The cultivar which recorded a significant incidence of pests and diseases was Clemson spineless, which was closely followed by Gombo Longbec and F1 Comet. Likewise, for the severity of pests and diseases, the most susceptible cultivar was Clemson Spineless, closely followed by Gombo Longbec. Meanwhile, the Landrace was the cultivar that recorded the least incidence and severity of pests and diseases.

The degree of incidence and severity of a cultivar to common pests and diseases is another important tool to determine its genetic potential (vigour) and adaptability. Meanwhile, Clemson spineless recorded significantly higher values for incidence and severity of pests and diseases, the Landrace on the contrary was more resistant to pests and diseases. This conforms with the findings of Dimkpa *et al*. [40] where Clemson spineless was the most susceptible cultivar to attack by *Podagrica* spp (okra flea beetle) and out of 7 cultivars under study, the least affected by pest and disease were the two landraces. Lamont [30] reported that *Abelmoschus caillei* (landrace) is prized for its yield, vigorous growth, and tolerant to negative environmental factors, serving as a source of many desirable characteristics” [41].

#### Yield components and yield of okra

F1 Comet and the Landrace recorded significant results for capsule length and capsule width respectively, while Kirikou recorded significant results for the number of capsules. Concerning yield, Kirikou recorded the most significant results and was closely followed by the Landrace. Rajesh *et al*. [42] found some variations in capsule length, width, number of capsules, capsule weight and yield of 14 okra accessions under study.

Crop yield is greatly influenced by the genetic potential of the cultivar, its adaptability to agroecological specificity and the adoption and implementation of best agronomic practices. The study recorded significant variability in terms of responses of the different cultivars to site-specificity vegetative growth parameters, incidence and severity of pests and diseases. Cultivars that recorded better results for vegetative growth parameters, as well as pest and disease resistance, also recorded better yields (i.e. Kirikou and the Landrace).

The Landrace due to its genetic potential and autogamous nature recorded optimal vigour and yield and was the most adaptable cultivar in terms of resistance to incidence and severity of pests and diseases. This implies that it could have genes of interest that can be useful in crop improvement, especially for those cultivars that were heavily infested with pests and diseases (i.e. Clemson spineless and Longbec). Tata Ngome *et al*. [25] had similar results in that some farmers preferred the “tall” (landrace) varieties of okra although they were late-maturing because they have a prolonged fruiting period spanning several seasons. Their study also reported that variability in varietal preferences could also be linked to taste. This is in agreement with the findings of this study as most farmers partly preferred the landrace for cooking because it tastes better, produces more mucilage (“draws” well) and a small quantity is required for cooking. In addition, since okra is autogamous, it is possible that there is little or no change (genetic modification) from one generation to another and this could play an important role in having more homozygous traits [43, 44]. This is possibly why farmers depend on the landrace since they have more or less 100% maintenance of these characters or traits, giving rise to a pure breed, as dominant or recessive characters are easily expressed in pure breeds than in heterozygous conditions.

#### Correlation of vegetative growth parameters and pest / disease severity with the yield of okra

Positive correlation values were recorded for the vegetative growth parameters, with stronger correlation for plant height and stem circumference with the yield. On the other hand pest and disease severity were inversely proportional to the yield and gave very low correlation values. Cultivars with significant vegetative growth and low infestation of pest and disease also recorded better adaptability. Pest and disease destroy important organs and tissues of the crop which in turn upsets the normal physiological functioning of the crop. For example Aphids and white flies are sucking pests of the leaves, while the Wilt destroys the vascular tissues of the crop rendering them inefficient in the uptake water and nutrients.

## Conclusion

The farmers’ system of okra seed supply and crop development in Buea which forms by far the most important source of seed in most farming systems in the world has been documented. Despite the efforts of large seed programmes to replace the farmers’ seed system, the major part of okra agricultural land in Buea is still sown with seeds that are informally gotten through mass selection by choosing the best capsules from two landraces identified as the 3-months (annual) and the 6-months (biennial) types. The storage methods used by farmers encouraged rapid deterioration in the quality, the reason why they had very high seeding rates.

Based on the adaptability test, Kirikou is the cultivar that was the most adaptable in Buea with a yield of 6.0 t/ha, followed by the Landrace (5.3t/ha). Early maturing cultivars included Clemson spineless and F1 Comet, medium maturing cultivars included Kirikou, Longbec, Landrace, Yellen and late-maturing cultivars were Cafeier and Nain.

This study has highlighted the need for coexistence of the formal and farmers’ seed systems and their improvement should be embraced, not only because they are mutually beneficial, but also since farmers and their communities cannot depend on one system only. The fundamental principle of the integrated seed sector development concept is the need to develop a twin-track approach, where the effectiveness of both the informal and the formal seed systems can be improved and through a concerted effort at every component of the seed value chain.

### Recommendation

For higher yields, farmers should plant cultivars like Kirikou, Landrace or F1 Comet. For farmers wanting quick returns, an early maturing cultivar such as F1 Comet or Clemson spineless can be planted.

## Supporting information

S1 File Vigor and yield. (XLSX)

S2 File Pest and disease severity. (XLSX)

S3 File Field survey. (XLSX)
